# Overexpression of MTA3 Correlates with Tumor Progression in Non-Small Cell Lung Cancer

**DOI:** 10.1371/journal.pone.0066679

**Published:** 2013-06-19

**Authors:** Haiying Li, Liangliang Sun, Ying Xu, Zixuan Li, Wenting Luo, Zhongping Tang, Xueshan Qiu, Enhua Wang

**Affiliations:** Department of Pathology, First Affiliated Hospital and College of Basic Medical Sciences, China Medical University, Shenyang, Liaoning, China; Ospedale Pediatrico Bambino Gesu’, Italy

## Abstract

The objective of the current study was to investigate the expression pattern and clinicopathological significance of MTA3 in patients with non-small cell lung cancer (NSCLC). The expression profile of MTA3 in NSCLC tissues and adjacent noncancerous lung tissues was detected by immunohistochemistry. MTA3 was overexpressed in 62 of 108 (57.4%) human lung cancer samples and correlated with p-TNM stage (p<0.0001), nodal metastasis (p = 0.0009) and poor prognosis (p<0.05). In addition, the depletion of MTA3 expression with small interfering RNAs inhibited cell growth and colony formation in the A549 and H157 lung cancer cell lines. Moreover, MTA3 depletion induced cell cycle arrest at the G1/S boundary. Western blotting analysis revealed that the knockdown of MTA3 decreased the protein levels of cyclin A, cyclin D1 and p-Rb. These results indicate that MTA3 plays an important role in NSCLC progression.

## Introduction

Lung cancer is one of the leading causes of all cancer-related deaths worldwide and its incidence is increasing [1], [2]. The majority of diagnosed lung cancer cases are non-small-cell lung cancers (NSCLCs). Although three therapeutic modalities (surgical resection, chemotherapy, and radiotherapy) have been established, the long-term survival of lung cancer patients is still generally poor [3]. A variety of complex genetic, epigenetic, and microenvironmental factors play important roles in the survival and colonization of tumor cells at new locations [4], [5]. Improvements in understanding molecular processes involved in pulmonary carcinogenesis has led to new treatment options with small molecules therapeutics and vaccines demonstrating encouraging potential. The better definition of lung cancer pathogenesis, useful biomarkers, and novel therapeutic targets are demanding tasks.

MTA3 was originally found as a member of a small protein family (including MTA1, MTA2 and MTA3), that all serve as subunits of the Mi-2/NuRD chromatin-remodeling complex [6]–[13]. Reports of MTA3 in human cancers were quite limited initially. MTA3 was reported to participate in B lymphocyte development, in plasmacytoma cell lines, the overexpression of BCL6 and MTA3 downregulated plasma cell differentiation genes [14]. Since then, however, the expression of MTA3 has found to be reduced in breast cancer, endometrial cancer and ovarian cancer [15]–[17]. MTA3 upregulation prevents EMT by directly repressing *Snail* expression, thereby upregulating E-cadherin protein levels in breast cancer [13]. MTA3 also represses Wnt4 transcription and secretion, inhibiting Wnt-target genes in mammary epithelial cells [18]. A recent study found that MTA3 facilitates G2/M progression in proliferating mouse granulosa cells [19]. Furthermore, MTA3 was reported as an independent and unfavorable prognostic marker in uterine non-endometriod carcinoma [20]. These studies have suggested different roles of MTA3 in different types of human cancers. However, the protein expression of MTA3 in primary lung cancer and its relationship with clinicopathological factors has not been examined. Therefore, the biological roles of MTA3 in lung cancer cells are still unclear. In order to address the above questions, we examined MTA3 protein expression in non-small-cell lung cancer tissues by immunohistochemistry. In addition, we explored the association of MTA3 with cell proliferation in several lung cancer cell lines.

## Materials and Methods

### Patients and Specimens

This study was conducted with the approval of the local institutional review board at the China Medical University. Written informed consent was obtained from all patients and all clinical investigations were conducted according to the principles expressed in the Declaration of Helsinki. 108 cases of NSCLC samples were obtained from the First Affiliated Hospital of China Medical University during the period of 2005 to 2008. The histological diagnosis and grade of tumor differentiation were defined through the evaluation of hematoxylin and eosin-stained tissue sections, according to classification guidelines of The World Health Organization. All 108 specimens were re-evaluated with respect to their histological subtypes, differentiation status, and tumor stages. For NSCLC samples, squamous cell carcinoma and adenocarcinoma were identified in 44 and 64 of the 108 cases, respectively. Lymph node metastases were observed in 43 patients. The p-TNM staging system of the International Union Against Cancer (7th Edition) was used to classify specimens in stages I (n = 47), II (n = 36), III (n = 25).

### Cell Lines

A549 and H157 cell lines were obtained from American Type Culture Collection (Manassas, VA, USA). The cells were cultured in RPMI 1640 (Invitrogen, Carlsbad, CA, USA) containing 10% fetal calf serum (Invitrogen), 100 IU/ml penicillin (Sigma, St. Louis, MO, USA), and 100 µg/ml streptomycin (Sigma). Cells were grown on sterile tissue culture dishes and passaged every 2 days using 0.25% trypsin (Invitrogen).

### Immunohistochemistry

Surgically excised tumor specimens were fixed with 10% neutral formalin, embedded in paraffin and 4 µm thick sections were prepared. Immunostaining was performed using the avidin–biotin–peroxidase complex method (Ultra Sensitive TM, Maixin, Fuzhou, China). The sections were deparaffinized in xylene, rehydrated in graded alcohol series and boiled in 0.01 M citrate buffer (pH 6.0) for 2 minutes in an autoclave. Endogenous peroxidase activity was blocked using hydrogen peroxide (0.3%), which was followed by incubation with normal goat serum to reduce non-specific binding. Tissue sections were incubated with a MTA3 rabbit polyclonal antibody (1∶150 dilution) (Proteintech, Chicago, IL, USA). Mouse immunoglobulin was used as a negative control. Staining with all primary antibodies was performed at room temperature for 2 h. Biotinylated goat anti-mouse serum IgG, or biotinylated goat anti-rabbit serum IgG(ready-to-use ) (Maixin, Fuzhou, China) was used as secondary antibodies. After washing, the sections were incubated with horseradish peroxidase-conjugated streptavidin–biotin, followed by 3,3′-diaminobenzidine tetrahydrochloride to develop the peroxidase reaction. Counterstaining of the sections was done with hematoxylin, and then ethanol dehydration was performed before mounting.

Two independent investigators examined all tumor slides randomly. Five views were examined per slide, and 100 cells observed per view at 400×magnification. Immunostaining of MTA3 was scored on a semi-quantitative scale by evaluating in representative tumor areas with a higher intensity and cell percentage of immunostaining than the control cells. Nuclear staining of the tumor cells was considered positive immunostaining. The intensity of MTA3 nuclear staining was also scored as 0 (no staining), 1 (weak), or 2 (marked). Percentage scores were assigned as 1 (1–25% positive), 2 (26–50%), 3 (51–75%), and 4 (76–100%). The scores of each tumor sample were multiplied to give a final score of 0 to 8 and the total expression of MTA3 was determined as either negative or low expression (−) with a score <4 or overexpression (+) with a score ≥4.

### Quantitative Real-time PCR (SYBR Green Method)

Quantitative real-time PCR was performed using SYBR Green PCR master mix (Applied Biosystems) in a total volume of 20 µl on 7900HT Fast Real-Time PCR System (Applied Biosystems) as follows: 95°C for 30 s, 40 cycles of 95°C for 5 s, and 60°C for 30 s. A dissociation step was performed to generate a melting curve to confirm the specificity of the amplification. β-actin was used as the reference gene. The relative levels of gene expression were represented as ΔCt = Ct gene –Ct reference, and the fold change of gene expression was calculated by the 2^−ΔΔCt^ method. Experiments were repeated in triplicate. The primer sequences used were:MTA3 forward, 5′-CCCACCCAGTCAGAAGAAGA-3′, MTA3 reverse, 5′-TTGGACTCCCAGTGTTTCG-3′; β-actin forward, 5′-ATAGCACAGCCTGGATAGCAACGTAC-3′, β-actin reverse, 5′-CACCTTCTACAATGAGCTGCGTGTG-3′; Snail forward, 5′-CCTCAAGATGCACATCCGAAGCCA-3′, Snail reverse, 5′-AGGAGAAGGGCTTCTCGCCAGTGT-3′; Slug forward, 5′-AGATGCATATTCGGACCCAC-3′, Slug reverse, 5′-CCTCATGTTTGTGCAGGAGA-3′.

### Western Blot Analysis

Total proteins from cell lines were extracted in lysis buffer (Thermo Fisher Scientific,Rockford,IL) and quantified using the Bradford method. Fifty micrograms of protein were separated by SDS–PAGE (12%). After transfer, the polyvinylidene fluoride (PVDF) membranes (Millipore, Billerica, MA, USA) were incubated overnight at 4°C with the following antibodies : MTA3(1∶1000; Proteintech, Chicago, IL, USA), beta-actin(1∶500; Santa Cruz Biotechnology, Santa Cruz,CA), cyclin A(1∶1000), cyclin B(1∶1000), cyclin D1(1∶1000), CDK2(1∶1000), CDK4(1∶1000), CDK6(1∶1000), and p-Rb(1∶2000) (Cell Signaling Technology, Boston, MA, USA). After incubation with peroxidase-coupled anti-mouse IgG and anti-rabbit IgG (Santa Cruz Biotechnology) at 37°C for 2 h, bound proteins were visualized using ECL (Thermo Fisher Scientific) and detected using BioImaging Systems (UVP Inc., Upland, CA, USA). The relative protein levels were calculated based on β-actin as the loading control.

### Small Interfering RNA Treatment

The siRNAs to MTA3 (siRNAa, 5′-CAGUGUAGAUUAUGUGCAATT-3′ and siRNAb, 5′-AGAUAAGCAUGCUAAAGAATT-3′) and negative control siRNAs (5′-UUCUCCGAACGUGUCACGUTT-3′) were purchased from Genepharma (Genepharma, Shanghai, China). For transfections, cells were seeded in a 24-well plate 24 h before the experiment. The cells were transfected with siRNAs using DharmaFECT 1 (0.20 µL/well; ThermoFisher Scientific) according to the manufacturer’s protocol. The mRNA and protein levels were assessed 48 h after transfection.

### Cell Proliferation Test and Colony Formation Assay

The cell proliferation assay was performed using Cell Counting Kit-8 solution (Dojindo, Gaithersburg, MD) according to the manufacturer’s protocol. Briefly, cells were seeded at a concentration of 5×10^3^ cells/100 µl/well in 96-well culture plates and treated with 10 µl/well of Cell Counting Kit-8 solution during the last 4 h of culture. The optical density of the well was measured at 450 nm using a microplate reader. For the colony formation assay, cells were planted into three 6-cm cell culture dishes (1000 per dish for A549 and H157 cell lines) and incubated for 12 days. Plates were washed with PBS and stained with Giemsa. Colonies with more than 50 cells were counted.

### Cell Cycle Analysis

Cells (500,000) were seeded into 6-cm tissue culture dishes. Twelve hours later, cells were transfected with the indicated amounts of siRNAs. Cells were synchronized after serum starvation for 20 h and time points taken at 24 h after incubation in 10% serum media to determine effects on the cell cycle. Cells were harvested, fixed in 1% paraformaldehyde, washed with phosphate-buffered saline (PBS) and stained with 5 mg/ml propidium iodide in PBS supplemented with RNase A (Roche, Indianapolis, IN) for 30 min at room temperature. Data were collected using BD Calibur flow cytometer.

### Statistical Analysis

SPSS version 16.0 for Windows was used for all analyses. The Chi-squared test was used to examine possible correlations between MTA3 expression and clinicopathological factors. The Kaplan-Meier method was used to estimate the probability of patient survival, and differences in the survival of patient subgroups were compared using Mantel’s log-rank test. The Student’s t-test was used to compare other data. The p value was based on the two-sided statistical analysis, and p<0.05 was considered to statistically significant.

## Results

### 1. Overexpression of MTA3 Protein in Non-small Cell Lung Cancer Tissues

**Figure 1:**
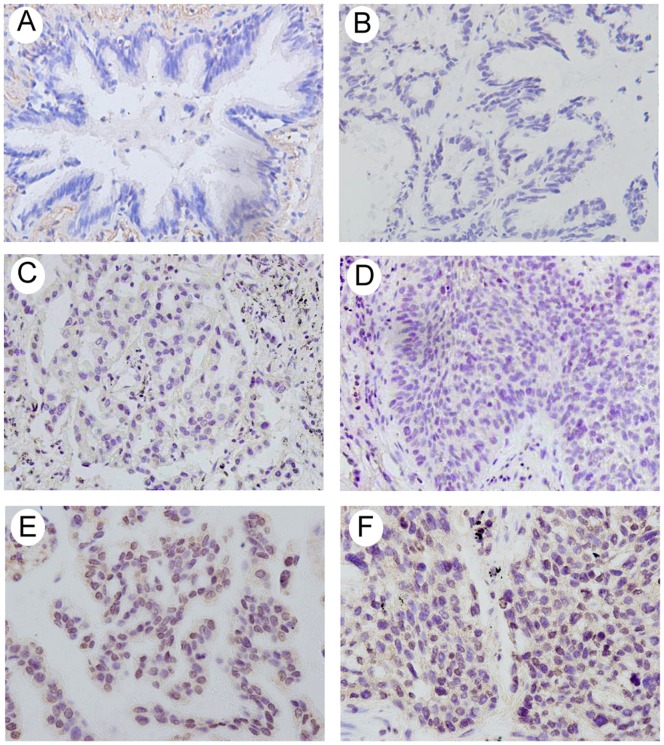
Immunohistochemical staining of MTA3 in lung cancer tissue sections. A. Negative staining in normal bronchial epithelium in non-cancerous lung tissue. B. Negative control using rabbit immunoglobulin. C. Weak MTA3 staining in lung adenocarcinoma. D. Weak MTA3 staining in a case of squamous cell carcinoma. E. Positive MTA3 staining in a case of lung adenocarcinoma. F. Positive MTA3 staining in a case of squamous cell carcinoma.

**Figure 2:**
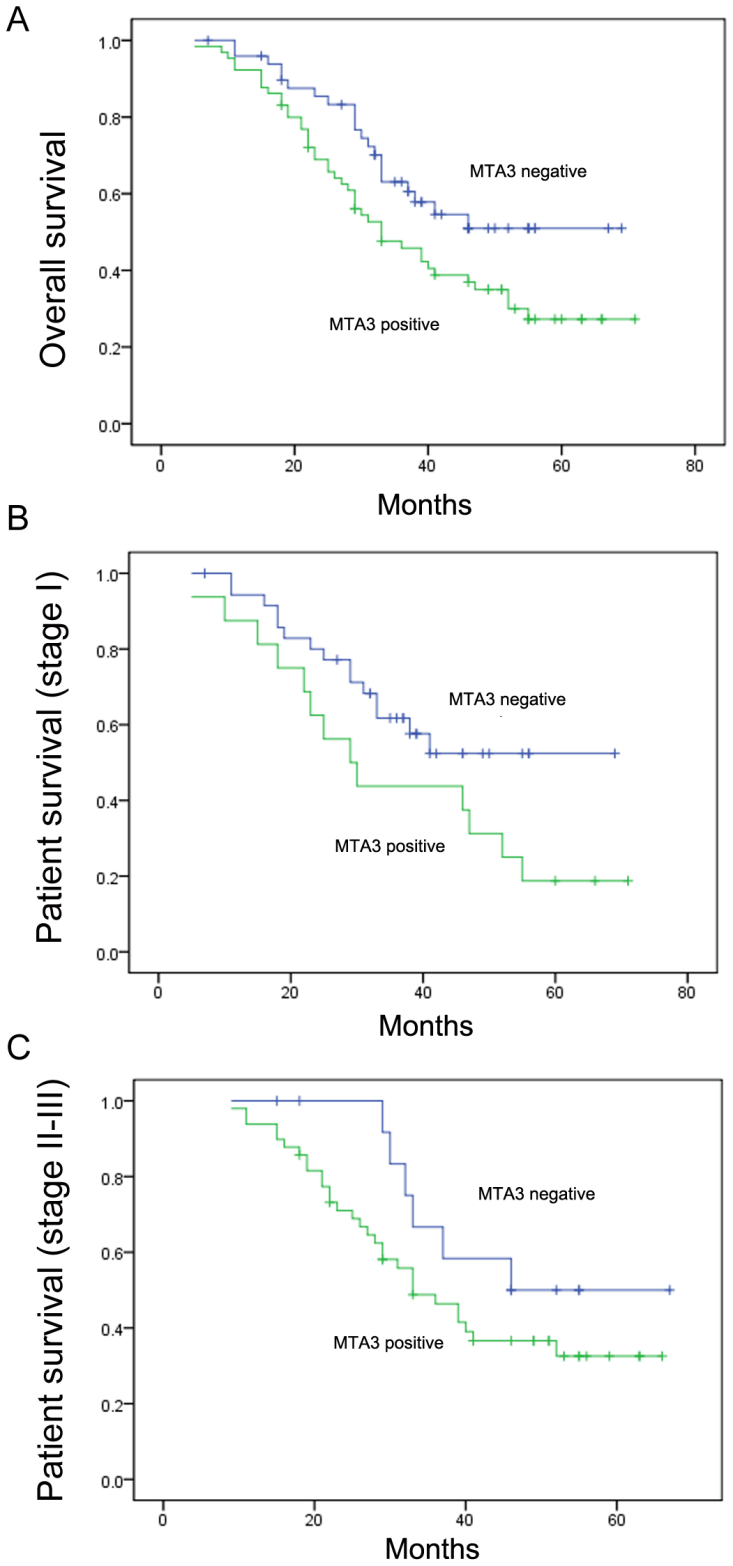
Survival analyses of patients with MTA3 overexpression. A. The overall survival rate was significantly lower in patients with positive MTA3 expression than in patients without. B. The survival rate in patients with stage I NSCLC. C. The survival rate in patients with stage II–III NSCLC.

We analyzed the protein expression of MTA3 in 108 NSCLC specimens and their corresponding normal tissues by immunohistochemistry. MTA3 protein expression was observed in the nuclear compartments of tumor cells, while the normal bronchial epithelia exhibited negative or low staining (Figure 1A–F). We investigated the relationship between total MTA3 expression and clinical parameters. As shown in Table 1, no statistical difference was found between MTA3 overexpression and characteristics of aging (p = 0.1404), gender (p = 0.8683), tumor status (p = 0.1556), differentiation (p = 0.1985) and tumor type (p = 0.0604). However, patients with high MTA3 expression showed increased nodal metastases (p = 0.0009) and had an advanced stage of NSCLC (p<0.0001). We further analyzed the relationship between MTA3 protein expression and the prognosis of lung cancer patients and found that MTA3 overexpression correlated with a reduction in overall survival (p<0.05) (Figure 2A). MTA3 overexpression correlated with the poor survival of stage I patients (p<0.05) but not of patients with stage II-III NSCLC (p = 0.17) (Figure 2 B, C).

**Table 1:** Distribution of MTA3 status in NSCLC according to clinicopathological characteristics.

Characteristics	Number of patients	MTA3negative	MTA3positive	*P*
Age				
<60	34	18(52.94%)	16(47.06%)	0.1404
≥60	74	28(37.84%)	46(62.16%)	
Gender				
Male	55	23(41.82%)	32(58.18%)	0.8683
Female	53	23(43.4%)	30(56.6%)	
Histology				
Adenocarcinoma	64	32(50%)	32(50%)	0.0604
Squamous cell carcinoma	44	14(31.82%)	30(68.18%)	
Differentiation				
Well	35	18(51.43%)	17(48.57%)	0.1985
Moderate- Poor	73	28(38.36%)	45(61.64%)	
TNM stage				
I	47	32(68.09%)	15(31.91%)	<0.0001
II	36	9(25%)	27(75%)	
III	25	5(20%)	20(80%)	
Tumor status				
T1	35	18(51.43%)	17(48.57%)	0.1556
T2	50	22(44.00%)	28(56.00%)	
T3 T4	23	6(26.09%)	17(73.91%)	
Nodal status				
N0	65	37(56.92%)	28(43.08%)	0.0009
N1	24	4(16.67%)	20(83.33%)	
N2 N3	19	5(26.32%)	14(73.68%)	

### 2. MTA3 Depletion Inhibits Proliferation in Lung Cancer Cell Lines

**Figure 3:**
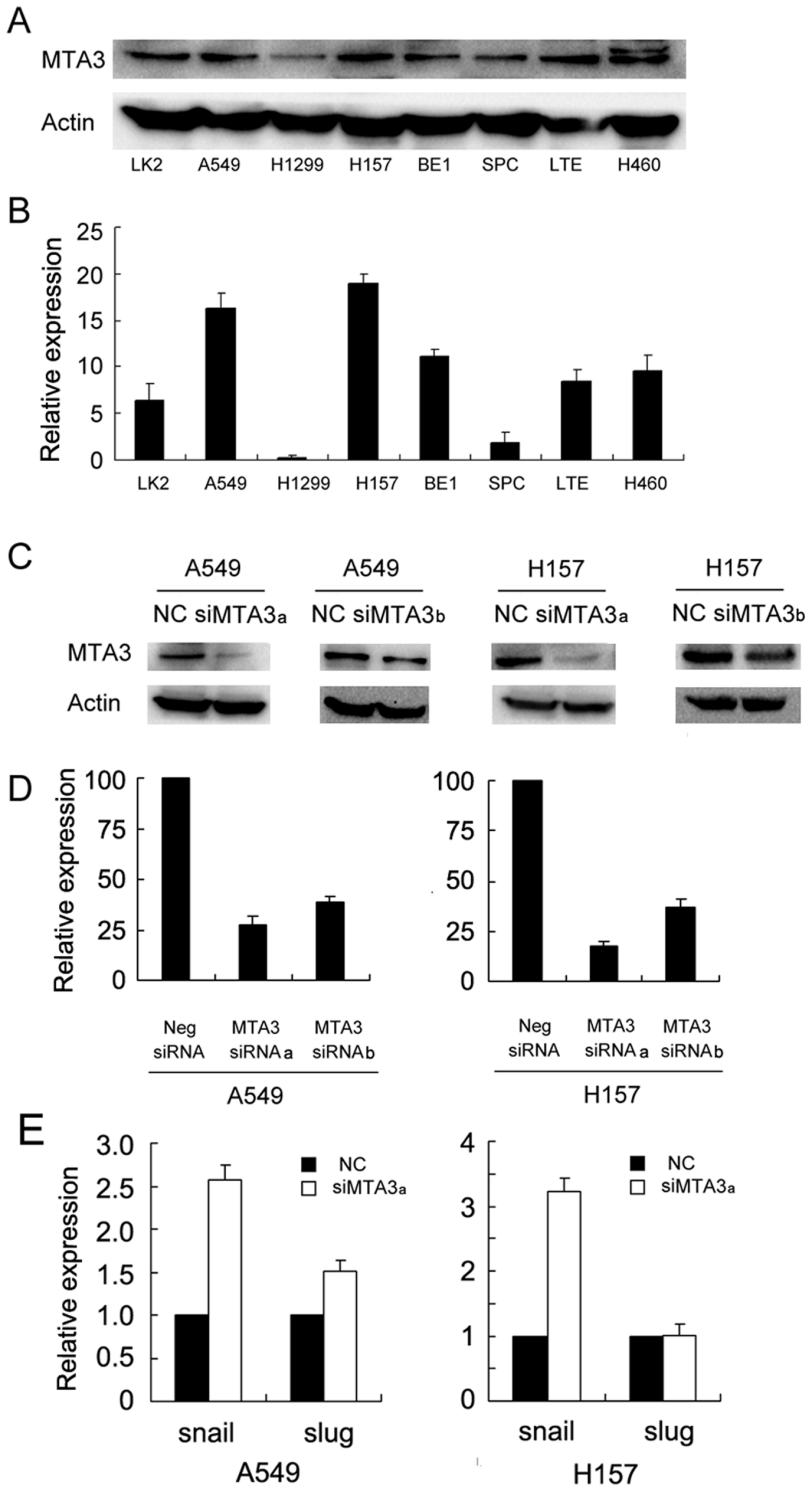
MTA3 depletion in A549 and H157 cell lines. A and B. Expression levels of MTA3 was analyzed by western blot and realtime PCR in a panel of lung cancer cell lines. C. Western blot analysis of two MTA3 siRNA efficiencies in cancer cells. D. Real-time PCR analysis of two MTA3 siRNA efficiencies in cancer cells. E. MTA3 siRNA treatment upregulated snail mRNA expression.

**Figure 4:**
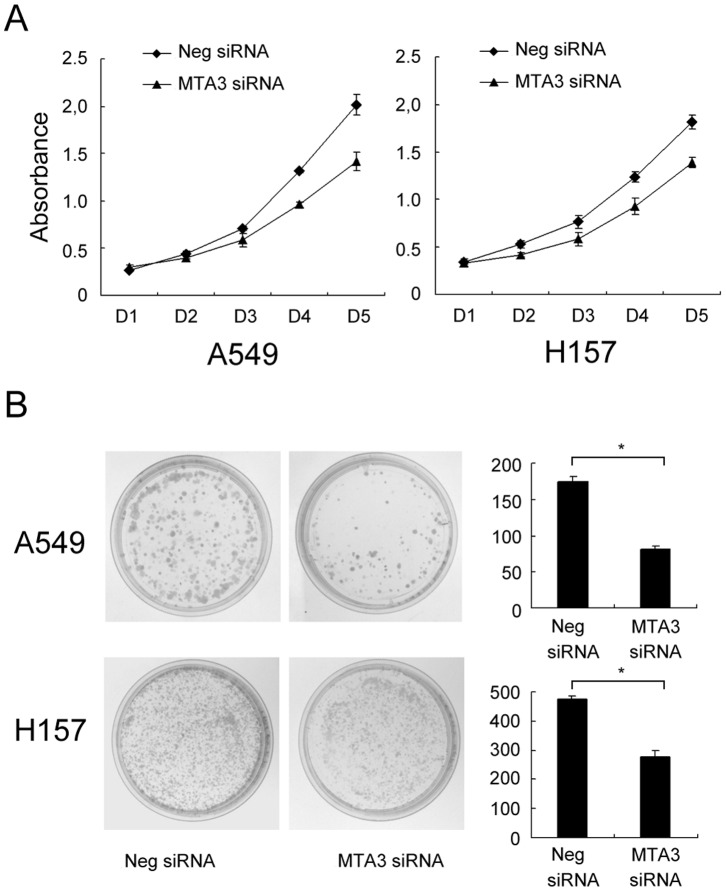
MTA3 depletion impaired cancer cell proliferation. A. CCK-8 assay was performed after MTA3 siRNA treatment. A reduction of absorbance was observed (p<0.05 at day 5 for both A549 and H157). B. Assessment of clonogenic potentials of the MTA3-depleted cancer cells. Numbers of colonies were counted. The number of colonies formed by cells treated with MTA3 siRNA was far less than that of control cells (p<0.05). Columns, mean; bars, SD. *p<0.05.

The expression of MTA3 was analyzed through Western blotting and realtime PCR in a panel of lung cancer cell lines (Figure 3 A, B). We found that the level of MTA3 expression in H157 and A549 cells was higher than other cell lines.To explore the biological function of MTA3 in lung cancer cells, we employed siRNA to knockdown *MTA3* expression in both H157 and A549 cell lines. Two MTA3 siRNAs (a and b), targeting different *MTA3* sequences, were evaluated. The siRNAa was more effective at reducing *MTA3* expression; thus we used it for further studies (Figure 3 C, D). To confirm the ability of siRNAa to downregulate MTA3 (and thus its transcriptional repression functions) we also examined the expression of the MTA3 downstream target genes *Snail* and *Slug*. As expected, treatment with MTA3 siRNAa upregulated the mRNA expression of *Snail* (Figure 3E). CCK-8 assay showed that MTA3 depletion reduced cell proliferaion in both cell lines. Colony formation analysis showed that the depletion of MTA3 in H157 and A549 cells led to a significant reduction in the number and size of foci (A549 control vs MTA3si: 275±7 vs 81±10; H157 control vs MTA3si: 476±10 vs 276±32), suggesting that MTA3 modulates the proliferation of lung cancer cells (Figure 4).

### 3. Depletion of MTA3 Downregulated Cyclina and Cyclin D1 Expression in Lung Cancer Cells

**Figure 5:**
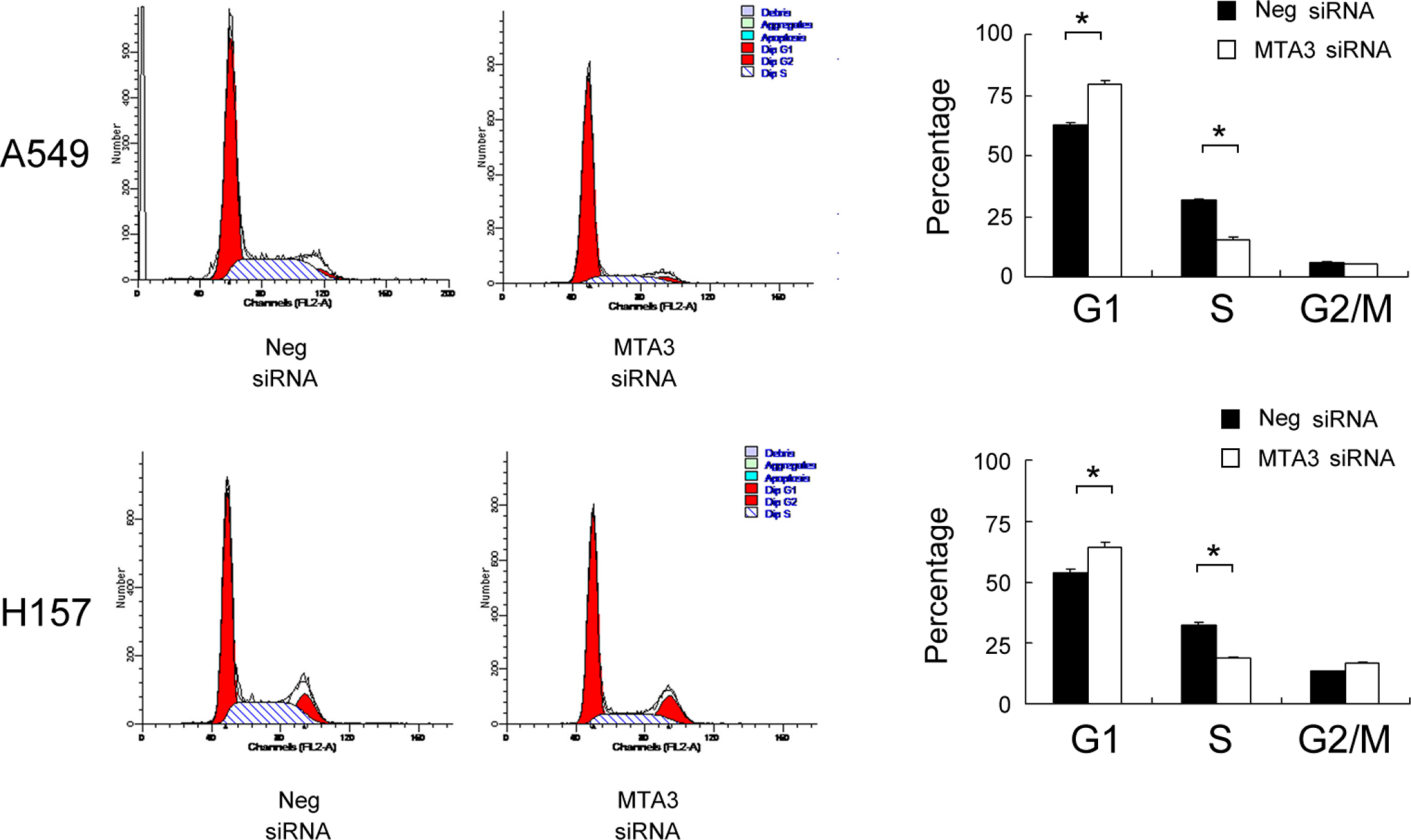
MTA3 knockdown inhibited cell cycle progression. The percentage of G1 phase was increased in cells with MTA3 knockdown (H157 and A549, p<0.05), whereas the percentages of S phase (H157 and A549, p<0.05) was decreased in these cells compared with control cells.

**Figure 6:**
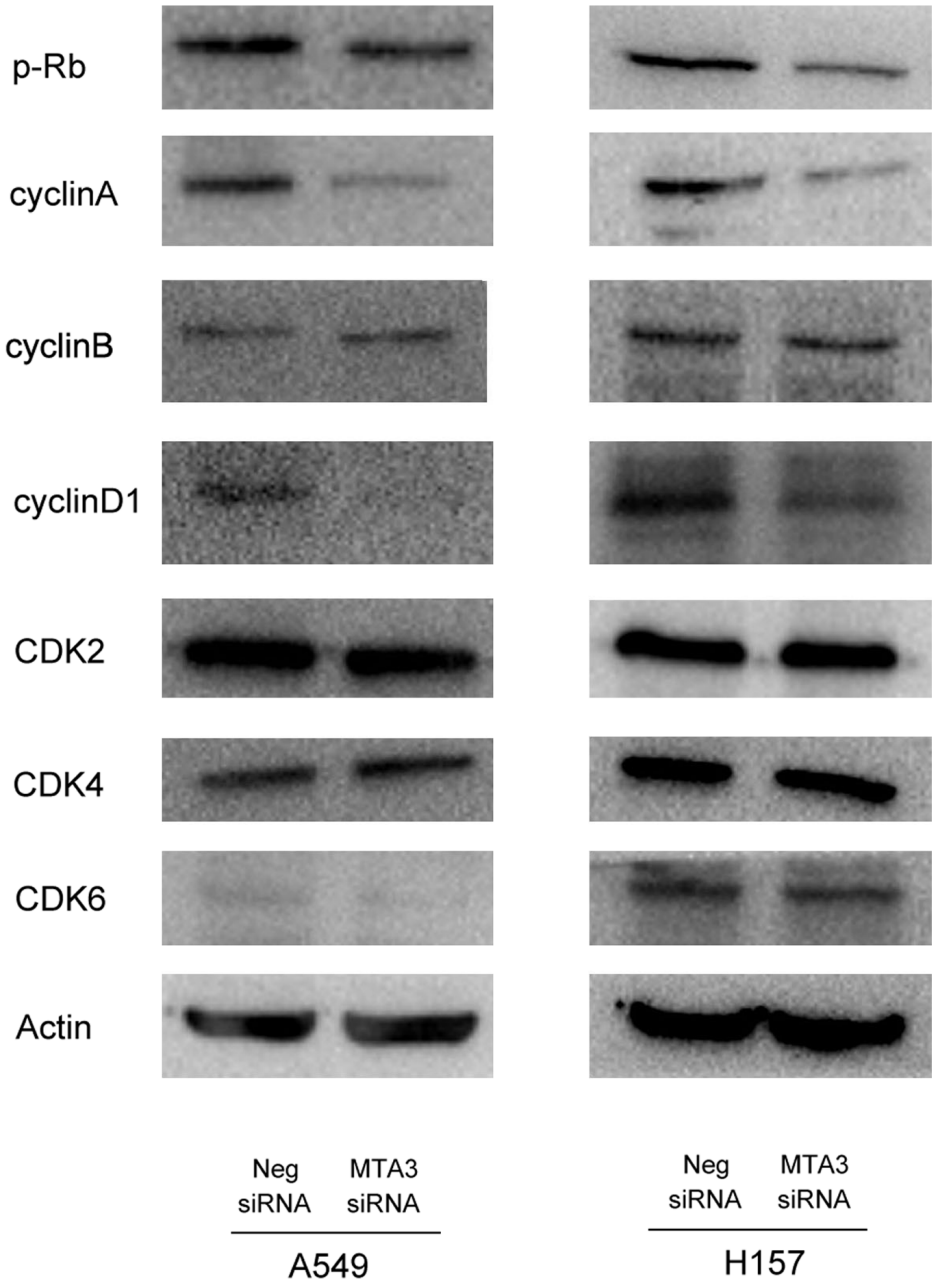
Expression of cell-cycle related molecules levels after MTA3 depletion. Western blot analysis of a series of cell cycle related factors showed the protein levels of Cyclin A, D1 and p-Rb were decreased after silencing MTA3 in H157 and A549 cells, while there was no significant change of Cyclin B, CDK2, CDK4 and CDK6 expression.

Cell cycle analyses were performed in A549 and H157 cells with or without MTA3 knockdown, and found that the percentage of cells in the G1 phase was increased in cells with MTA3 knockdown, whereas the percentage of cells in the S phase decreased in these cells compared with control cells(A549 control vs MTA3si: G1 phase: 62.59%±0.9 vs 79.71%±1.5; S phase: 31.27%±0.52 vs 15.21%±0.88; H157 control vs MTA3si: G1 phase: 53.83%±1.4 vs 64.61%±1.8; S phase: 32.64%±0.8 vs 18.59%±0.48). These results indicate that the depletion of MTA3 induces cell cycle arrest at the G1/S boundary. There was no significant change in the proportion of G2 phase cells (Figure 5). To investigate the mechanism underlying cell cycle arrest, we examined the levels of the cycle-related proteins using cells harvested at the same time point as cells used in the cell cycle analysis. We tested the effects of MTA3 knockdown on the levels of cyclin A, cyclin D1, cyclin B, CDK2,CDK4, CDK6 and p-Rb. Western blotting analysis revealed that the knockdown of MTA3 decreases the protein levels of cyclin A, cyclin D1 and p-Rb expression (Figure 6). Together, these results suggest that inhibiting MTA3 expression induces cell cycle arrest at the G1-S transition, suppressing lung cancer cell growth.

## Discussion

The downregulation of MTA3 was reported in breast cancer, endometrial cancer and ovarian cancer [15]–[17], while the upregulation of MTA3 expression has been implicated in human chorionic cancer [21]. MTA3 overexpression serves as a prognostic marker for judging the survival of uterine non-endometriod cancer patients [20]. However, the expression pattern of MTA3 as well as its correlation with clinical and pathological factors had not yet been defined in human lung cancer. In this study, we demonstrated that MTA3 protein expression in human lung tissues is higher than corresponding normal lung tissues. There was a close correlation between MTA3 upregulation pTNM stage and nodal metastasis. Importantly, we were able to show that MTA3 correlates with the poor survival of lung cancer patients. This was in accordance with previous data that has suggested that MTA3 plays an important role in lung cancer progression. To validate the potential role of MTA3 in lung cancer development, we first checked its expression level in several cell lines and used A549 and H157 cells (with relatively high MTA3 levels) for further studies. We employed siRNA to knockdown MTA3 expression in these two cell lines. We found an impaired proliferation capacity and colony formation ability in both cell lines after MTA3 knockdown. Thus, our study suggests that *MTA3* functions as an oncogene in lung cancer development.

A rencent study reported that t the depletion of endogenous MTA3 in mouse primary granulosa cells significantly decreases cyclin B1 and cyclin B2 expression, slows cell proliferation and increaseds the percentage of cells in G2/M, which could be reversed by the co-expression of exogenous MTA3 [19]. Most proliferative factors influence cell growth by affecting cell cycle progression. Thus we employed cell cycle analysis and found an increase in MTA3-deficient cells in the G1 phase and a decrease in the S phase compared to control cells. The inhibition of the G1 to S transition in cell cycle progression might explain the mechanisms behind MTA3 effects on lung cancer cell proliferation.

To find the potential mechanisms of MTA3 on cell cycle regulation, we examined the effect of MTA3 knockdown on a number of cell-cycle-related molecules. We checked the expression of cyclins (A, B, and D1), CDK2, CDK4, CDK6 and p-Rb. We found that the levels of cyclinA, D1 and p-Rb decreased after MTA3 knockdown. Cyclin D1 interacts with Cdk4/6 to form a complex that phosphorylates Rb and regulates cell proliferation by controlling progression through the restriction point within the G1-phase of the cell cycle [22]. Cyclin D1 is overexpressed in a variety of cancers and is associated with cancer cell proliferation [23]–[25]. Cyclin A is required for the cell to progress through S phase [26]. Cyclin B is a mitotic cyclin and its accumulation is only found at the G2-M transition [27]. Thus, our results showing decreased levels of cyclin A, D1, and p-Rb correlate with the decreased levels of cells in the S and G2 phase and the increased levels of cells in the G1 phase after MTA3 knockdown, suggesting MTA3 plays an important role in cell cycle control of lung cancer cells.

In conclusion, the present study showes that MTA3 is overexpressed in NSCLC and correlates with its advanced stage and poor prognosis. Our study also demonstrates that the overexpression of MTA3 might promote cell proliferation through cell cycle regulation.
